# Stroke Exacerbates Cancer Progression by Upregulating LCN2 in PMN-MDSC

**DOI:** 10.3389/fimmu.2020.00299

**Published:** 2020-02-21

**Authors:** Tingting Huang, Yan Li, Yuxi Zhou, Bingwei Lu, Yueman Zhang, Dan Tang, Yu Gan, Zhengzhou He, Zengai Chen, Weifeng Yu, Peiying Li

**Affiliations:** ^1^Department of Anesthesiology, Renji Hospital, School of Medicine Shanghai Jiaotong University, Shanghai, China; ^2^State Key Laboratory of Oncogenes and Related Genes, Shanghai Cancer Institute, Renji Hospital, School of Medicine Shanghai Jiaotong University, Shanghai, China; ^3^Department of Radiology, Renji Hospital, School of Medicine Shanghai Jiaotong University, Shanghai, China

**Keywords:** stroke, cancer, myeloid derived suppressed cell, lipocalin 2, neutrophil (PMN), myeloid cell

## Abstract

Acute ischemic stroke (AIS) is common in patients with cancer, and mounting clinical evidence suggests that it may shorten the survival of cancer patients. But how stroke affects the progression of cancer remains unclear. We inoculated B16 tumor cells (2 × 10^5^) subcutaneously before distal middle cerebral artery occlusion (dMCAO) or sham surgery in C57BL/6 mice and found that compared to sham operated mice, dMCAO mice developed significantly increased tumor volume and were accompanied by lower survival rate. To explore the underlying mechanism, we performed RNA-sequencing analysis of the tumor tissue from mice with or without stroke and found prominent upregulation of lipocalin 2 (LCN2) in the tumor from stroke mice compared to those from sham mice. Using quantitative reverse transcription-PCR, we confirmed increased mRNA expression of LCN2 as well as anti-inflammatory cytokines-Arg1, IL-10, and decreased mRNA level of pro-inflammatory cytokines-IL-6, IL-23 in the tumor of cancer-bearing stroke mice. Both immunofluorescence staining and flow cytometry analysis revealed that increased expression of LCN2 was mainly derived from the polymorphonuclear myeloid derived suppressor cells (PMN-MDSCs) in the tumor. We also found that stroke reduced the PMN-MDSCs in the peripheral blood, but increased PMN-MDSCs in the tumor of the cancer-bearing mice after stroke. In conclusion, cerebral ischemic stroke may exacerbate cancer progression by increasing LCN2 expression in PMN-MDSCs, which turns out to be a promising therapeutic target to suppress cancer progression after ischemic stroke.

## Introduction

Cancer is the leading cause of death and a major public health threat all over the world (1). There's increasing evidence that active cancer increases short-term risk of stroke, and ischemic stroke can be the first complication of systemic cancer (2). Furthermore, overall survival after stroke in patients with cancer has been shown to be shorter. The median survival in cancer bearing stroke patients is only 4.5 months from the diagnosis of stroke (3). Despite of mounting evidence suggesting exacerbated cancer progression after stroke, it remains largely unknown the underlying mechanism how the acute ischemic stroke event alters the progression of cancer.

Myeloid-derived suppressor cell (MDSC) is a specific immune suppressive cell population that has been acknowledged to mediate immunologic tolerance in tumor microenvironment (TME), such as promoting tumor cell survival, angiogenesis, invasion of healthy tissue by tumor cells, and metastases (4). There are two major types of MDSC, polymorphonuclear MDSC (PMN-MDSC) that are similar to neutrophils, whereas monocytic MDSC (M-MDSC) similar to monocytes in morphology and phenotype. Despite the fact that MDSCs share the same origin and differentiation pathways, these cells have distinct features and biological roles in a large array of pathologic conditions ranging from cancer to obesity (5). PMN-MDSCs are pathologically activated in many diseases, such as renal cell carcinoma (6), colorectal cancer (7), glioblastoma (8), and etc. These cells are pivotal for the regulation of immune responses in cancer, and their presence are associated with poor prognosis and negative responses to immunotherapy (8, 9). However, their roles in the context of cancer-bearing stroke remain undefined.

In this study, we found that acute ischemic stroke induced by distal middle cerebral artery occlusion (dMCAO) in B16 melanoma mice model significantly exacerbated the cancer progression. Using RNA-sequencing, we found 18 genes were significantly upregulated in the tumor of cancer-bearing stroke mice compared to that in the cancer mice without stroke, among which, LCN2, an adipokine, which was previously suggested to play important roles in cancer progression exhibited significant increase in the mRNA level. Both flow cytometry and immunofluorescence suggested that ischemic stroke induced LCN2 expression in PMN-MDSC in the tumor and promoted the recruitment of PMN-MDSC into the tumor, which may underlie the increased immune suppressive microenvironment of the tumor after stroke. These findings suggest that PMN-MDSC derived LCN2 may potentially serve as an immune modulatory target for the exacerbated cancer progression in cancer-related stroke.

## Methods

### Mice and Tumor Model

All animal experiments were approved by the Renji Hospital Institutional Animal Care and Use Committee and performed in accordance with the Institutional Guide for the Care and Use of Laboratory Animals. To establish tumor model, B16F10 cells (2 × 10^5^/mice) suspended in 100 μL DMEM were injected subcutaneously in the left flanks of male 6- to 8-week old C57BL/6J mice (10). Permanent distant middle cerebral artery occlusion (dMCAO) surgery was performed at 12–14 days when the average tumor volume had grown to 800–1,300 mm^3^. The tumor volume was measured with caliper and calculated by the formula for ellipsoid (V = length × width^2^ × π/6).

### Mice Model of Cerebral Ischemic Stroke

Male 6- to 8-week old C57BL/6J mice were purchased from Shanghai SLAC Laboratory. Focal cerebral ischemia was generated by permanent dMCAO, plus ipsilateral common carotid artery (CCA) occlusion. To begin with, mice were anesthetized with 3% isoflurane in 67%:30% N_2_O/O_2_ (induction), until they were unresponsive to the tail pinch test and then fitted with a nose cone providing 1.5% isoflurane for anesthesia maintenance. Neck skin was incised at the midline and the left CCA exposed and ligated. After the neck incision was sutured, the midline of skin incision in left eye and ear was made. The temporal muscle was dissected, and a bone window opened to expose the middle cerebral artery (MCA). The dura matter was then cut and the distal MCA was coagulated with low-intensity bipolar electrocautery at the immediate lateral part of the rhinal fissure. Rectal temperature was controlled at 37.0 ± 0.5°C during surgery and MCA occlusion via a temperature-regulated heating pad. Animals were randomly assigned to sham and dMCAO groups through the use of a lottery-drawing box. Sham-operated animals underwent the same anesthesia and surgical procedures but were not subjected to dMCAO and CCA occlusion (11).

### RNA Sequencing and Bioinformatics of Transcriptomes

RNA samples were prepared from the tumors tissues of cancer-only, cancer + stroke (3 animals/group). RNA sequencing was performed using BGISEQ-500. Briefly, we prepared the libraries starting with 1 μg total RNA for each sample. After cDNA first strand synthesis, we amplified the product by 15 cycles. We then carried out the second size selection operation and selected 103–115 bp fragments from the gel. This step was conducted in order to purify the PCR product and remove any non-specific products. After gel purification, we quantified the PCR yield by Qubit (Invitrogen; Cat No. Q33216) and pooled samples to make a single strand DNA circle (ssDNA circle), which gave the final miRNA library. DNA nanoballs (DNBs) were generated with the ssDNA circle by rolling circle replication (RCR) to enlarge the fluorescent signals at the sequencing process. The DNBs were loaded into the patterned nanoarrays and single-end reads of 50 bp were performed on the BGISEQ-500 platform for the following data analysis study. For this step, the BGISEQ-500 platform combines the DNA nanoball-based nanoarrays and stepwise sequencing using polymerase. The new modified sequencing approach provides several advantages, including among others, high throughput and quality of patterned DNB nanoarrays prepared by linear DNA amplification (RCR) instead of random arrays by exponential amplification (PCR) as, e.g., used by Illumina's HiSeq and longer reads of polymerase-based cycle sequencing compared to the previously described combinatorial probe-anchor ligation (cPAL) chemistry on DNB nanorrays. The use of linear DNA amplification instead of exponential DNA amplification generates sequencing array results with lower error accumulation and sequencing bias.

Differentially expressed genes (DEGs) were analyzed to assess enrichment for GO, KEGG using NOISeq package. By calculating the average expression of the gene in the Control group and the treatment group, we obtained the difference multiple (MA = log2((treaty-avg)/(control-avg)) and the deviation probability value of the gene, and screened out the differentially expressed gene according to the default standard of difference multiple ≥2 and deviation probability value ≥0.8. There are three ontologies (ontologies) in GO (Gene Ontology), which, respectively, describe the molecular function of genes, cellular component, and biological process. GO function enrichment analysis gives the GO function items that are significantly enriched in DEGs compared with the genomic background, thus indicating which biological functions are significantly correlated with differentially expressed genes. The analysis first put all the differentially expressed genes to the Gene Ontology database of each term mapping, calculate each term number of genes, then using hypergeometric inspection, find out compared with the entire genome background, significantly enriched in the differentially expressed genes GO entries. For pathway analysis, we identified the major biochemical metabolic pathways and signal transduction pathways involving DEGs through pathway significant enrichment based on KEGG Pathway and hypergeometric test, *Q*-value ≤ 0.05.

### Flow Cytometry

For splenocytes and peripheral blood cells, single cell suspensions were prepared using RBC lysis buffer (BD) and filtered through a 70 μm nylon membrane. For tumor tissues, single cell suspensions were prepared using the tumor dissociation kit (Miltenyi Biotec, 130-096-730) according to the manufacturer's recommendations. Antibodies specific for the mouse cell surface markers CD45-PE-A (catalog: 25-0451-82, ebioscience, USA), CD11b-APC (catalog: 101212, BD), Ly-6G-BV421 (catalog: 1127628C, BD, USA), Ly6C-PE-Cy7 (catalog: 128018, BD, USA), LCN2-Alexfluo488 (catalog: ab63929, Abcam, Britain), NK1.1-PE-A (catalog: 12-5941-81, ebioscience, USA), CD3-APC-A (catalog: 17-0032-80, ebioscience, USA), CD11c-APC-A (catalog: 17-0114-82, ebioscience, USA), F4/80 (catalog: 4341619, Invitrogen, USA).

### Immunofluorescence Staining and Confocal Imaging

Separate animal groups of cancer-only and cancer-stroke mice were euthanatized at indicated time-points after dMCAO. Tumors were removed following perfusion with saline and 4% paraformaldehyde (Sigma-Aldrich, St Louis, MO) in phosphate buffered saline (PBS) and then cryoprotected in 30% sucrose in PBS. They were cut on a freezing microtome into 20 μm-thick sections and subjected to immunofluorescence staining. Primary antibodies included rabbit anti-LCN2 (catalog: ab63929, Abcam, Britain), mouse anti-Gr1 (catalog: BE0075, Biocell, USA), mouse anti-F4/80 (catalog: 123140, BD, USA). Images were processed with Image J for counting of automatically-recognized cells.

### Quantitative Real-Time Polymerase Chain Reaction

In brief, total tumor RNA was extracted using Trizol. The first stand of cDNA was synthesized with 1 μg RNA using the HiScript III RT SuperMix Kit (Vazyme). Quantitative real-time polymerase chain reaction was performed on the Lighter Cycle 480 II (Roche) using ChamQ™ SYBR Color qPCR Master Mix (Vazyme). Primers used are as follows: LCN2 forward primer: 5′-TGGCCCTGAGTGTCATGTG-3′, reverse primer: 5′-CTCTTGTAGCTCA TAGATGGTGC-3′; Arg1 forward primer: 5′-CTCCAAGCCAAAGTCCTTAGAG-3′, reverse primer: 5′-AGGAGCTGTCATTAGGGACATC-3′; iNOS forward primer: 5′-GTTCTCAGCC CAAC AATACAAGA-3′, reverse primer 5′-GTGGACGGGTCGATGTCAC-3′; IL-6 forward primer: 5′-CCAAGAGGTGAGTGCTTCCC-3′, reverse primer: 5′-CTGTTGTTCAGACTCT CTCCCT-3′; IL-23 forward primer: 5′-ATGCTGGATTGCAGAGCAGTA-3′, reverse primer: 5′-ACGGGGCACATTATTTTTAGTCT-3′; IL-10 forward primer: 5′-GCTCTTACTGACTGGCA TGAG-3′, reverse primer: 5′-CGCAGCTCTAGGAGCATGTG-3′. Expression of β-Actin mRNA served as an internal control.

### Statistical Analysis

Continuous data are presented as means ± standard error (SEM). Data with two groups were analyzed using two-tailed Student's *t*-test. Data with three or more groups were analyzed with one-way ANOVA and Bonferroni *post-hoc* test was performed for multiple comparisons. Differences in means across multiple groups with multiple measurements over time were analyzed using two-way ANOVA. In all analyses, *p* ≤ 0.05 was considered statistically significant. GraphPad Prism software (version 5.0) was used for statistical analyses.

## Results

### Acute Ischemia Stroke Exacerbates Cancer Progression

**Figure 1:**
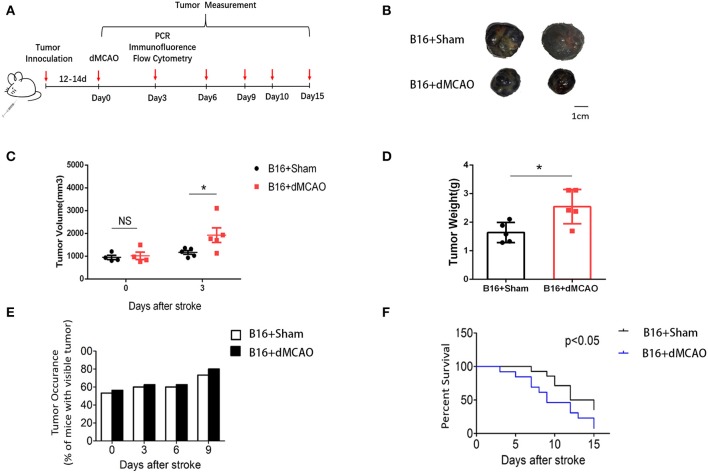
Cerebral ischemic stroke accelerates cancer progression. **(A)** Schematic representation of the experimental design. **(B)** Representative images of tumors collected from mice of each group. **(C)** Tumor volume measured at indicated time points after stroke (*n* = 4–6 in each group, *P ≤ 0.05). **(D)** Quantification of tumor weights of the above tumors collected at 3 days after stroke (*n* = 6 in each group, *P ≤ 0.05). **(E)** Percentage of tumor occurrence in mice with visible tumor among mice inoculated with B16 cell line (*n* = 6 in each group). **(F)** Overall survival analysis of mice in the two experimental groups up to 15 days after stroke (*n* =13 in sham group, *n* = 14 in stroke group, *P ≤ 0.05).

To examine how acute ischemic stroke affect cancer progression, we first induced dMCAO in B16 inoculated melanoma mice model (Figure 1A). We measured tumor volume and weight at 0, 3, 6, and 9 days after stroke and found that tumors in stroke mice grew substantially larger than those in mice without stroke (Figures 1B–D). Besides, we observed that mice developed tumors at around 10 days after implantation and stroke mice showed higher visible tumor than control mice (Figure 1E). Survival analysis demonstrated that cancer-stroke mice had significantly lower survival rate compared to cancer-only mice (Figure 1F). These results suggest that ischemia stroke substantially exacerbates cancer progression and leads to poor prognosis.

### RNA-Sequencing Reveals Transcriptional Alterations in Tumors After Stroke

**Figure 2:**
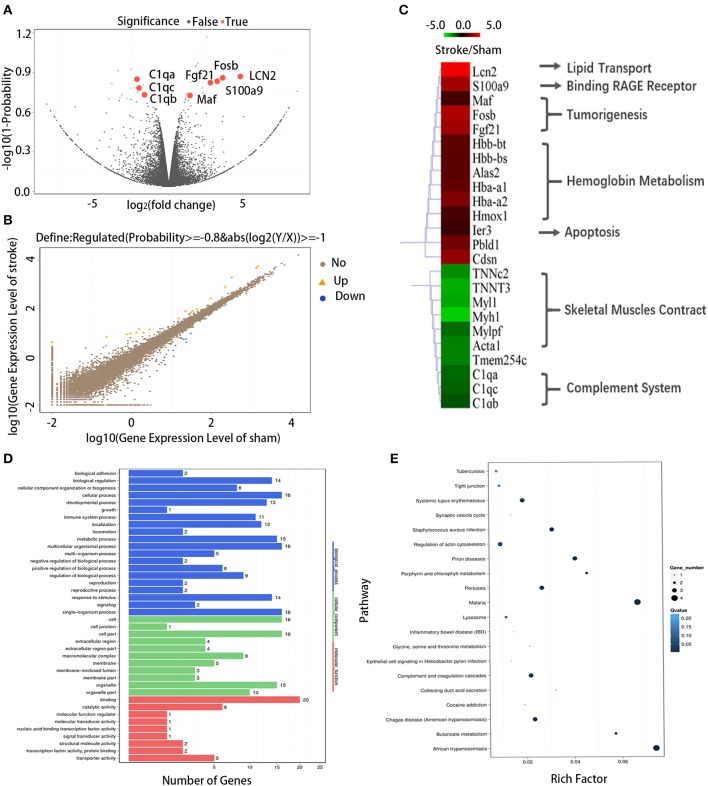
Differentially expressed genes between the dMCAO and sham group. **(A)** DEGs displayed on a volcano plot. Red spots represent genes whose expression was significantly changed between the dMCAO and sham group. Gray spots represent genes without significant changes. **(B)** DEGs displayed on a scatter plot. Blue and orange spots represent genes that were 2-fold decreased or increased in the tumor of dMCAO mice, respectively (*n* = 3/group, *P* ≤ 0.05). Brown spots represent genes without significant different changes. **(C)** Hierarchical clustering of the DEGs. Green and red represent genes that were 2-fold decreased or increased in the tumor of dMCAO mice, respectively (*P* ≤ 0.05). **(D)** GO enrichment analysis results for all DEGs. **(E)** KEGG pathway enrichment analysis results for all DEGs. dMCAO, Distant middle cerebral artery occlusion; DEG, Differentially expressed gene; GO, Gene Ontology; KEGG, Kyoto Encyclopedia of Genes and Genomes.

To explore whether acute ischemic stroke induces alterations in the transcriptional profile of the tumor, we performed RNA sequencing analysis of the tumors collected from cancer mice 3 days after sham or dMCAO surgery. Differential mRNA expressions between the dMCAO and sham operated mice were represented in volcano and scatter plots (Figures 2A,B). As is shown on Figure 2B, 18 genes were significantly up-regulated and 13 genes were downregulated in melanoma tissues of dMCAO mice compared to those in sham mice. Hierarchical clustering of differentially-expressed genes (DEGs) showed that acute ischemic stroke induced upregulation of genes included *Lcn2, Snord104, Rn7s2, Fam177a, Hbb-bs, Cdsn, Hbb-bt, Alas2, Hmox1, Pbld1, Fgf21, Mir5136, Hba-a2, S100a9, Hba-a1, Ier3, Fosb, Trib3* but down-regulation of genes included *C1qc, Cd74, Mylpf, Atp6v0c-ps2, Acta1, C1qa, Myl1, Tnnt3, Myh1, Tnnc2, Tmem254c, Hmga1-rs1, C1qb*. (Figure 2C). Gene Ontology (GO) enrichment analysis was performed with DEGs (Figure 2D). The top 5 enriched GO biological processes were binding, cell part, single-organism process, multicellular-organism process, metabolic process. The DEGs between the dMCAO and sham mice were enriched to 25 subclasses of pathways in 5 broad categories (cellular processes, human diseases, organismal systems, metabolism, and environmental information processing) when analyzed using the KEGG database (Figure 2E). The top 5 pathways with the greatest enrichment were malaria, African trypanosomiasis, prion diseases, pertussis and complement and coagulation cascades, indicating a compromised immune response in the tumor after acute ischemic stroke. Among the 18 upregulated genes identified in the RNA sequencing, lipocalin-2 (LCN2) not only exhibited high expression level, with the FPKM value of 6.68, but also was increased by 4.84 folds in the stroke mice compared to the sham mice. Considering that LCN2 is an important player of the tumor microenvironment and it has been suggested to be critical for cancer progression (12), we focused on in our following experiments.

### Stroke Induces Upregulation of LCN2 and an Immunosuppressive Cytokine Production in the Tumor

**Figure 3:**
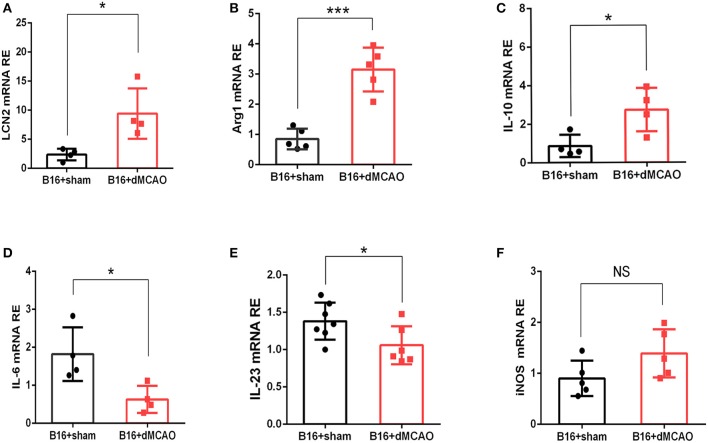
Stroke induces increased mRNA expression of LCN2 and anti-inflammatory Arginase1, and IL-10 in the tumor. **(A–C)** The mRNA expression of LCN, Arg1, and IL-10 **(A–C)** was increased in the tumor 3 days after stroke (*n* = 4–6, *P* ≤ 0.05). **(D,E)** The mRNA expression of IL-6 and IL-23 was decreased in the tumor from cancer stroke mice compared with cancer sham mice (*n* = 4–6, *P* ≤ 0.05). **(F)** The mRNA expression level of iNOS remained not significantly changed after stroke in cancer mice (*n* = 4–6, *P ≤ 0.05, ***P ≤ 0.001, two-tailed Student's *t*-test). Data are presented as the mean ± SD. LCN2, Lipocalin 2; Arg1, Arginase 1; iNOS, Inducible nitric oxide synthase.

In order to validate our findings in the RNA sequencing experiment, and examine whether the mRNA level of LCN2 could be altered by stroke, RNA were extracted from tumor tissues and quantitative real-time polymerase chain reaction (RT-PCR) was performed. As shown in Figure 3A, the relative expression of LCN2 was significantly higher in the tumor from stroke mice than that from sham mice, which is consistent with the previous RNA-seq results. We next examined the mRNA expression of some pro-inflammatory and anti-inflammatory cytokine to evaluate the immune responses in the tumor from the two mice groups. As is shown in Figures 3B–F, the mRNA expression of anti-inflammatory cytokines Arg1 and IL-10 were dramatically increased (Figures 3B,F) whereas the mRNA of pro-inflammatory cytokines IL-6 and IL-23 were significantly down-regulated (Figures 3D,E). Although the mRNA expression of iNOS exhibited an increased tendency, there was no statistical significance (Figure 3C). Together, these results demonstrate that acute ischemic stroke induces upregulation of the mRNA level of LCN2 and an immunosuppressive cytokine production in the tumor.

### LCN2 Is Increased in the PMN-MDSCs in the Tumor After Stroke

**Figure 4:**
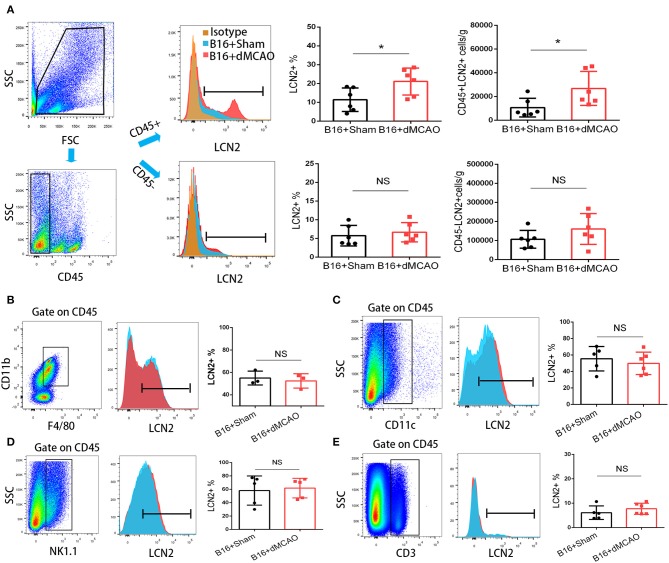
Increased expression of LCN2 in CD45^+^ cells in the tumor of stroke mice. **(A)** Gating strategy of the flow cytometry of the tumor sample from cancer mice 3 days after stroke or sham surgery. Upper right panels showed increased LCN2^+^CD45^+^ cells in the tumor of cancer mice after stroke. Lower panels revealed no significant difference in the LCN2^+^CD45^−^ cells (cancer cells) in the tumor of stroke mice. **(B,C)** LCN2 expression in CD11b^+^CD45^+^F4/80^+^ cells (tumor associated macrophages, TAMs) and CD11c cells (Dendritic cells) in the tumor. **(D,E)** LCN2 expression in NK1.1 cells (NK cells) and CD3^+^ cells (T cells) in the tumor (*n* = 3–6/group. *P ≤ 0.05).

**Figure 5:**
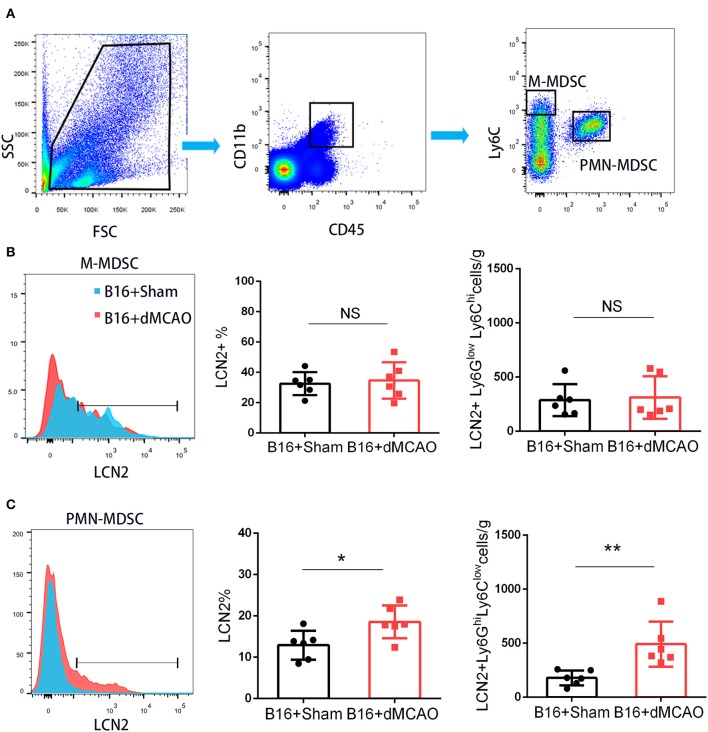
PMN-MDSC may be the major source of the increased LCN2 in the tumor after stroke. **(A)** Gating strategy of the MDSC populations in the tumor 3 days after sham or stroke surgery. **(B,C)** LCN2 expression in M-MDSC (CD45^+^CD11b^+^Ly6G^low^Ly6C^hi^) and PMN-MDSC (CD45^+^CD11b^+^Ly6G^hi^Ly6C^low^) in the tumor tissues after stroke (*n* = 6/group. *P ≤ 0.05, **P ≤ 0.01).

**Figure 6:**
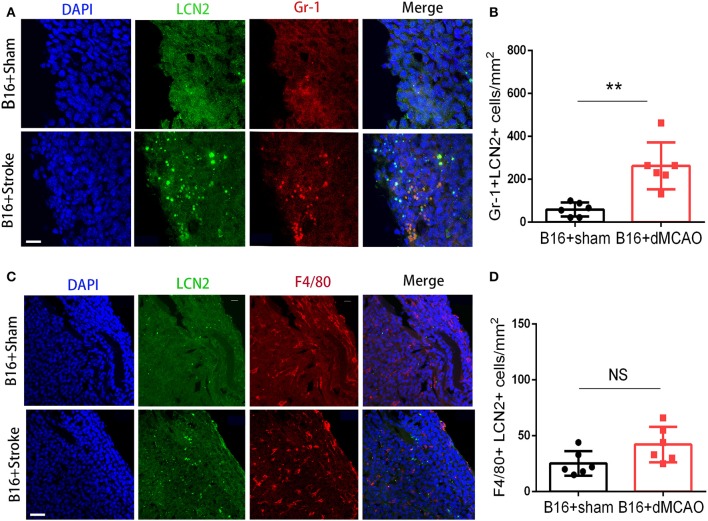
LCN2 expression in Gr1^+^ and F4/80^+^ cells in the tumor. Tumor tissue were collected for immunofluorescence staining 3 days after sham or stroke surgery. **(A,B)** Representative images and quantification of LCN2 and Gr1 double staining in the tumor after stroke. Scale bar =10 μm. *N* = 6/group. **(C,D)** Representative images and quantification of LCN2 and F4/80 double staining in the tumor after stroke. Scale bar = 20 μm (*n* = 6/group. **P ≤ 0.01).

In order to elucidate the cellular source of the increased LCN2 in the tumor, we first performed flow cytometry of the tumor tissue from mice with or without stroke. We found that the LCN2^+^ cells among CD45- cells, which are mainly tumor cells, did not change significantly after acute ischemic stroke (Figure 4A). Instead, the percentage of LCN2^+^ cells among CD45^+^ cells and the number of LCN2^+^CD45^+^ cells were significantly increased in the tumor after stroke, suggesting that the increased LCN2 were mainly derived from leukocytes (Figure 4A). Given that tumor microenvironment (TME) is composed of tumor-associated macrophages (TAM), polymorphonuclear myeloid-derived suppressor cells (PMN-MDSC), monocytic MDSC (M-MDSC), and dendritic cells (DC), and etc., we analyzed the LCN2 expression in the CD11b^+^, F4/80^+^, CD11c^+^, NK1.1^+^, and CD3^+^ cells in the tumor of the cancer mice with or without stroke. We found that the LCN2 expression in these cells were not changed significantly in the tumor after stroke (Figures 4B–E). Since MDSCs are abundant in tumor and play important roles in tumor progression (13), we then investigated whether LCN2 expression in M-MDSC (CD11b^+^Ly6G^low^Ly6C^hi^) and PMN-MDSC (CD11b^+^Ly6G^hi^Ly6C^low^) were changed in the tumor after stroke. We found no significant difference in the percentage of LCN2^+^ cells among M-MDSCs or the number of LCN2^+^CD11b^+^Ly6G^low^Ly6C^hi^ cells between the two groups (Figure 5). However, the percentage of LCN2^+^ cells among PMN-MDSCs or the number of LCN2^+^ CD11b^+^Ly6G^hi^Ly6C^low^ cells were significantly increased in the tumor after stroke (Figure 5). Next, we confirmed the upregulation of LCN2 expression in the tumor using immunofluorescence staining. We found that there are significantly more LCN2^+^ Gr1^+^ cells in the tumor of stroke mice than that in the sham mice (Figures 6A,B). However, the LCN2^+^F4/80^+^ cells did not change significantly between the groups (Figures 6C,D). Collectively, the above data suggests that ischemic stroke induces increased LCN2 expression in the PMN-MDSCs in the tumor of cancer-bearing mice.

### Stroke Reduced MDSCs in the Peripheral Blood in the Cancer-Bearing Mice

**Figure 7:**
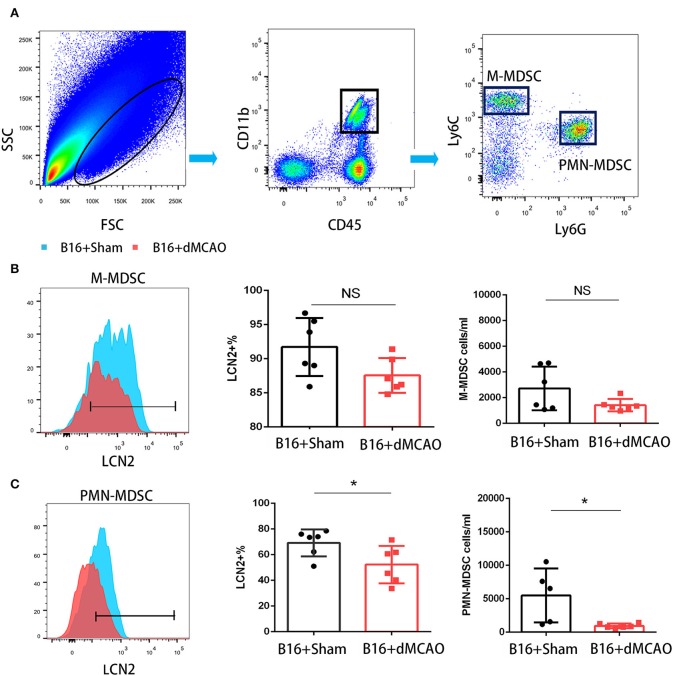
Flow cytometry of LCN2 expression in M-MDSCs and PMN-MDSCs from blood. **(A)** Gating strategy of the MDSC populations in blood 3 days after sham or stroke surgery. **(B,C)** LCN2 expression in M-MDSC (CD45^+^CD11b^+^Ly6G^low^Ly6C^hi^) and PMN-MDSC (CD45^+^CD11b^+^Ly6G^hi^Ly6C^low^) in the blood after stroke (*n* = 6/group. *P ≤ 0.05).

Using flow cytometry, we also examined the MDSCs in the peripheral blood in the cancer-bearing mice 3 days after stroke or sham surgery. We found that compared to the sham mice, the stroke mice had significantly fewer LCN2^+^ CD45^+^CD11b^+^Ly6G^hi^Ly6C^low^ cells and LCN2^+^ CD45^+^CD11b^+^Ly6G^low^Ly6C^hi^ cells in the peripheral blood (Figure 7). The percentage of LCN2^+^ cells among PMN-MDSCs were also decreased in the stroke mice compared to the sham mice (Figure 7). However, the percentage of LCN2^+^ CD45^+^CD11b^+^Ly6G^low^Ly6C^hi^ cells did not change significantly in the peripheral blood of cancer-bearing mice after ischemic stroke (Figure 7).

## Discussion

This study addressed that ischemic stroke could exacerbate cancer progression. The gene expression profile could change significantly in the tumor of stroke mice compared to that in the sham mice. The increased LCN2 expression in the tumor of stroke mice was derived from PMN-MDSC in the tumor. This is the first study to explore the gene transcriptional changes of cancer in the context of ischemic stroke using RNA sequencing. Our findings suggest that the recruitment of PMN-MDSC and the increased expression of LCN2 might contribute to the enhanced immune suppressive tumor microenvironment thus aggravate cancer progression.

Recently, cancer-related stroke is attracting increasing attention in clinical research. Mounting epidemiology studies suggest that cancer-bearing patients may have increased cancer progression after acute ischemic stroke, which is very common in cancer patients (14). However, it remains largely unknown how acute ischemic stroke exacerbate cancer progression in the cancer-related stroke. Using RNA sequencing, we profiled the gene expression in the tumor of cancer-bearing stroke mice compared to that of cancer-bearing sham mice, and found 18 genes were significantly upregulated in the cancer-bearing stroke mice. Among these genes, we noticed that LCN2 exhibited high expression level and significant upregulation in the stroke mice compared to the sham mice.

LCN2 is an adipocyte fatty acid binding protein that can bind and transport small lipophilic substances such as retinoids, arachidonic acid, and various steroids (15). Arachidonic acid metabolism has been considered to play a key role in carcinogenesis (16). LCN2 has also been shown to promote malignant progression in many cancer types and can serve as a biomarker for cancer patients. It has also been suggested to be involved in the regulation of proliferative cells (17). By RNA-sequencing of the tumor tissue from cancer mice with or without stroke, we identified that the mRNA expression of LCN2 was significantly increased in the tumor after stroke. We also observed that the increased LCN2 in the tumor were mostly derived from those recruited PMN-MDSCs in the tumor. PMN-MDSCs are a subset of MDSCs that can contribute to tumor malignancy by inhibiting immune responses and promoting tumor angiogenesis, tumor cell invasion, metastasis, etc. (18). Mounting evidence has demonstrated that there are cross-talks between MDSC and T cells, macrophages, dendritic cells, NK cells in TME (19), all of which may result in an immunosuppressive tumor microenvironment. Lipocalin-2 also shows increased level in various human diseases such as inflammation, infection, and ischemia (20). Characterized as an inflammatory protein and iron regulator, LCN2 is deemed as a new biomarker in neurodegenerative diseases (21). As is discovered, LCN2 can be expressed in various components of the CNS, including neurons, the choroid plexus, microglia, and astrocytes in pathological conditions and serves as a “help-me” signal to activate astrocytes and microglia, facilitating neurovascular recovery after stroke and brain injury and mediating anti-inflammatory effects against sepsis-induced brain damage and behavioral changes (22). Interestingly, LCN2 has also been found to induce proinflammatory cytokines as well as inducible nitric oxide synthase, which might trigger secondary damage and hinder recovery (23). However, the comprehension of its roles in pathology of stroke, especially in the context of cancer is far from complete.

Here we observed that acute cerebral ischemic stroke reduced the number of PMN-MDSC in the peripheral blood and increased the recruitment of PMN-MDSCs in the tumor. As we know that the recruitment of immune cells into tumor is regulated by multiple factors, including the micro-environment of the tumor (24). Previous studies have investigated that neutrophils and PMN-MDSC are recruited primarily by CXC chemokines which incorporate CXCL1, CXCL5, CXCL6, CXCL8, and CXCL12 while monocytes and M-MDSCs recruited by CCL2 (25). S100A8 and S100A9 proteins are also regarded as signals to recruit PMN and PMN-MDSC to pre-metastatic sites in colon cancer (26). After cerebral ischemic stroke, the ischemic brain can also release danger-associated molecular patterns (DAMPs), such as high mobility group box1 [HMGB1; (27)]. Some of the alarmins could induce profound alterations of the peripheral immune system, leading to mobilization and even exhaustion of peripheral immune cells (28, 29). Blocking the stroke-induced immune disturbance may not only attenuate ischemic brain injury, but also prevent post-stroke infection (30). In the context of cancer-bearing stroke mice, the immune disturbance should exacerbate ischemic brain injury by paradoxical recruitment of immune suppressive cells into the tumor (11). As an important immune suppressive subset, MDSCs have been strongly linked to the immune suppressive micro-environment and cancer progression. In the current study, we found that acute ischemic stroke induced increased expression of LCN2 in the tumor. PMN-MDSC could be the major source of the increased LCN2 in the cancer-bearing stroke mice. We further found that the PMN-MDSC in the tumor was increased, and the PMN-MDSC in the peripheral blood was decreased. However, the mechanism that underlies the changes of the PMN-MDSC in the blood and tumor in the context of stroke and caner still remain to be further explored.

In conclusion, we find that ischemic stroke exacerbates cancer progression and leads to the upregulation of a number of genes in the tumor of stroke mice compared to the sham mice. The expression of LCN2 in the PMN-MDSC in the tumor were significantly increased in stroke mice compared to that in sham mice, and it may potentially serve as an immune modulatory target for the exacerbated cancer progression in cancer-related stroke.

## Data Availability Statement

The raw data supporting the conclusions of this article will be made available by the authors, without undue reservation, to any qualified researcher.

## Ethics Statement

The animal study was reviewed and approved by Renji Hospital Institutional Animal Care and Use Committee.

## Author Contributions

TH, YL, YZho, BL, and YZha performed the experiments. DT, YG, ZH, and ZC collected the data and performed the analysis. TH, YL, and PL wrote the manuscript. YG and PL designed the experiments. WY and PL supervised the project.

### Conflict of Interest

The authors declare that the research was conducted in the absence of any commercial or financial relationships that could be construed as a potential conflict of interest.
